# Transoesophageal echocardiography of anomalous origin of the right coronary artery from the pulmonary artery

**DOI:** 10.1093/ehjcr/ytae182

**Published:** 2024-04-09

**Authors:** Lu Li, Hui Yang

**Affiliations:** Department of Anesthesiology, West China Hospital, Sichuan University, No.37 Guoxue rode, Wuhou District, Chengdu 610041, China; Department of Anesthesiology, West China Hospital, Sichuan University, No.37 Guoxue rode, Wuhou District, Chengdu 610041, China

## Case description

**Figure 1:**
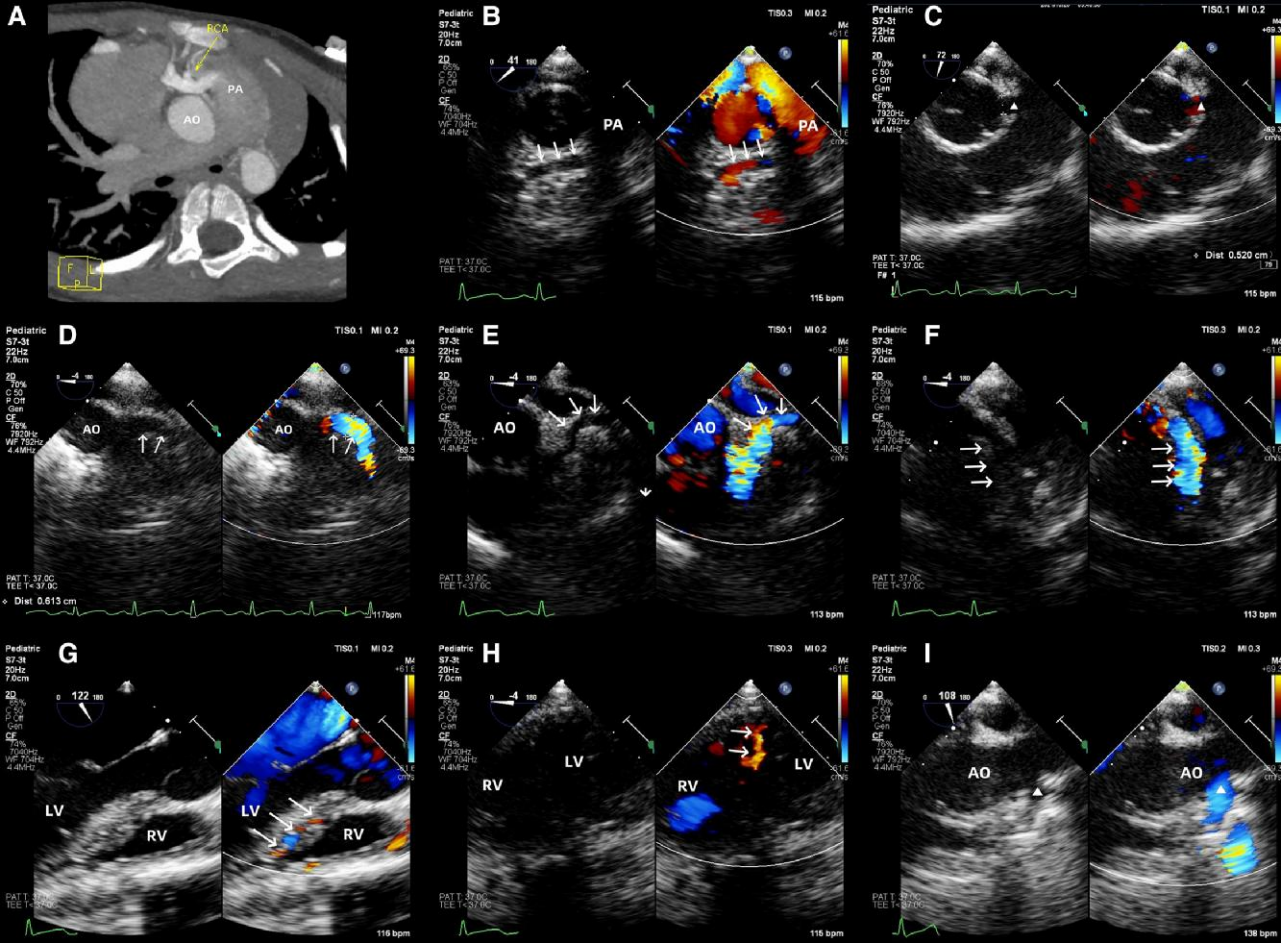
Transoesophageal echocardiography was utilized to assess an anomalous origin of the RCA from the MPA. (*A*) CCTA reveals the RCA arising from the MPA (arrow). (*B*) In the mid-oesophageal aortic valve short axis view, the RCA is observed emerging from the MPA (arrows). (*C*) Mid-oesophageal aortic valve short axis view shows the dilated LCA arising centrally from the left sinus of Valsalva (triangle). Based on mid-oesophageal five-chamber view, the dilated LMCA (*D*) was visualized by slight adjustment of the probe. Following the course of the LMCA, the dilated LCX (*E*) and LAD (*F*) are identified (arrows). (*G*) Mid-oesophageal long axis view shows flow signals within the ventricular septum (arrows). (*H*) Transgastric mid-short-axis view demonstrates flow signals within the ventricular septum (arrows). (*I*) Post-operative TEE examination reveals the re-implanted RCA in the mid-oesophageal aortic valve long axis view (triangle). AO, aorta; RCA, right coronary artery; PA, pulmonary artery; LV, left ventricle; RV, right ventricle; CCTA, coronary computer tomography angiography; LCA, left coronary artery; LMCA, left main coronary artery; LCX, left circumflex coronary artery; LAD, left anterior descending artery.

A 4-year-old male child was admitted for pneumonia, exhibiting symptoms of cough and sputum production, but not experiencing dyspnoea, chest pain, or palpitations. During physical examination, a grade 2/6 systolic ejection murmur was detected at the left upper sternal border. Transthoracic echocardiography (TTE) revealed an anomalous connection between the right coronary artery (RCA) and the main pulmonary artery (MPA), along with dilation of the left coronary artery (LCA). This finding was further confirmed by coronary computer tomography angiography (CCTA), which showed the RCA arising directly from the MPA (*Figure 1A*). The child was diagnosed with anomalous origin of the right coronary artery from the pulmonary artery (ARCAPA). Due to the potential risk of myocardial ischaemia or sudden death, surgical correction was performed after the pulmonary infection was effectively controlled. Prior to the procedure, a transoesophageal echocardiogram (TEE) was performed to reconfirm the diagnosis and evaluate cardiac function. The TEE results indicated that the LCA originated from the left sinus of Valsalva (*Figure 1C*), with both the left anterior descending (LAD) and left circumflex (LCX) arteries showing dilation and tortuosity (*Figure 1E* and *F*). The RCA was found to originate from the MPA rather than the right sinus of Valsalva (*Figure 1B*). The ejection fraction was measured at 63% with no wall motion abnormalities. The child underwent coronary artery re-implantation, excision of the RCA button, and transfer to the ascending aorta using the punch hole technique. Post-operative TEE confirmed successful re-implantation with good biventricular function and proper coronary flow (*Figure 1I*). The child was discharged four days after surgery.

Anomalous origin of the right coronary artery from the pulmonary artery is a rare condition in clinical practice, with an incidence rate of approximately 0.002%.^1^ Around 70–75% of ARCAPA cases are isolated, wherein patients typically exhibit no evident clinical symptoms.^2^ However, the pressure difference between the coronary arteries and pulmonary arteries can lead to coronary steal, resulting in myocardial ischemia, acute heart failure, and even sudden death.^3^ Anomalous origin of the right coronary artery from the pulmonary artery is a life-threatening congenital disease, therefore, surgical intervention is recommended for all diagnosed cases, even if the patient is asymptomatic.Anomalous origin of the right coronary artery from the pulmonary artery, a rare disease usually asymptomatic, is commonly discovered incidentally during examinations for other medical conditions. Transoesophageal echocardiogram is a dependable diagnostic tool for ARCAPA, although its effectiveness can be limited by operator experience. Recognizing typical echocardiographic manifestations of ARCAPA is crucial to minimize missed or incorrect diagnoses. These manifestations include a normal origin of the LCA with a widened internal diameter and increased flow velocity. The origin of the RCA is not visualized in the right sinus of Valsalva but is instead located in the PA. Moreover, colour Doppler echocardiography reveals extensive collateralization with flow signals within the ventricular septum, as well as a shunt from the LCA to the PA. Intra-operative TEE can supplement preoperative information, assist surgical decision-making, and evaluate surgical effects in real time. It is a reliable and effective diagnostic and monitoring tool.

## Data Availability

The data underlying this article are available in the article.
